# The Cellular and Molecular Characteristics of Postnatal Human Thymus Stromal Stem Cells

**DOI:** 10.3390/biomedicines13041004

**Published:** 2025-04-21

**Authors:** Josipa Skelin Ilic, Ildikó Bódi, Lidija Milkovic, Zsolt Prodan, Dražen Belina, Darko Heckel, Lipa Cicin-Sain, Danka Grčević, Domenico Vittorio Delfino, Delfa Radic Kristo, Maja Matulić, Mariastefania Antica

**Affiliations:** 1 Division of Molecular Mediicne, Rudjer Boskovic Institute, 10000 Zagreb, Croatia; 2 Department of Anatomy, Histology and Embryology, Semmelweis University, 1085 Budapest, Hungary; 3 Kids Heart Center Budapest, 1096 Budapest, Hungary; 4 Department of Cardiac Surgery, University Hospital Centre, 10000 Zagreb, Croatia; 5 School of Medicine, University of Zagreb, 10000 Zagreb, Croatia; 6 Foligno Nursing School, Department of Medicine and Surgery, University of Perugia, 06034 Foligno, Italy; 7 Section of Pharmacology, Department of Medicine, University of Perugia, 06123 Perugia, Italy; 8 Department of Biology, Faculty of Science, 10000 Zagreb, Croatia; 9 Institute for Medical Research and Occupational Health, 10000 Zagreb, Croatia

**Keywords:** human thymus, epithelial stem cell, 3D thymus culture

## Abstract

**Background**: The thymus is the central hub of T-cell differentiation, where epithelial cells guide the process of their maturation. **Objective**: Our goal was to identify and describe progenitor cells within the human thymus that can differentiate into epithelial cells. **Methods**: When we plated enriched thymic cells in 3D culture conditions, rare individual cells capable of self-renewal and differentiation formed spheroids. **Results**: Both neonatal and adult thymuses produced similar numbers of spheroids, suggesting that progenitor potential remains consistent across age groups. Some cells within the spheres express genes typical of mature epithelial cells, while others express genes associated with the immature compartment active during thymic organogenesis. However, there were also cells expressing PDGFRβ. We treated the tissues with 2-deoxyguanosine before digestion, which improved the yield of progenitor cells. We also cultured the enriched stromal thymocytes with Cyr61 and Interleukin-22, which affected the spheroid size. **Conclusions**: Our efforts towards thymic reconstitution are ongoing, but our research uncovers previously unknown characteristics of the elusive epithelial progenitor population.

## 1. Introduction

The adaptive immune system, the development, maturation and function of T lymphocytes, depends on the thymus. Thymic cortical (cTEC) and medullary (mTEC) epithelial cells, along with other cells of the stromal compartment, guide the differentiation and the selection of competent T lymphocytes through a complex multi-step process involving tests of functionality, sensitivity and self-tolerance [1,2]. With ageing, the thymus goes through a process of involution, during which epithelial cells are replaced by connective tissue, fibroblasts and adipocytes [3,4]. In addition to this natural process, the thymus is extremely sensitive to damage caused by chemo-, radio- or antibodytherapy. The degeneration of the stromal compartment inevitably leads to a loss in the number of naive T-cells produced and a decreased ability to fend off infections [5,6]. The search for an epithelial progenitor/stem cell capable of differentiating into both cortical and medullary epithelial cells needed for T-cell selection has been the focus of the field. Some groups have found that the adult thymus contains self-renewing, bipotent epithelial progenitors. However some studies dispute this, suggesting that the 3D spheroids originate from the mesenchymal lineage and do not support T-cell development [7,8,9]. Most of the published work focuses on the mouse model, which we cannot completely translate to humans, and both mouse and human thymic stromal progenitor cells remain to be precisely characterized. Here we show, on a substantial number of postnatal samples, that the human thymus, indeed, contains rare progenitor/stem cells with a spheroid-forming and self-renewal capacity, expressing genes involved in early thymic epithelial development as well as those typical for mature thymic epithelial cells.

## 2. Materials and Methods

### 2.1. Thymus Tissue

All of the thymuses used in experimental procedures were obtained from children who underwent a thymectomy as a part of the heart surgery at the University Hospital Centre Zagreb. The oldest patient from whom a thymus was obtained was 10 years old. However, we use the term “adult thymus”, as the thymus is considered to be fully developed right after birth in humans. Written informed consent was given by the children’s guardians in accordance with the Helsinki Declaration of 1975, as revised in 2008. The ethics committees from both the Rudjer Boskovic Institute (approval number BP-5865/1-2013) and the University Hospital Zagreb (Class: 8.1.-13/88-2, approval number: 02/21-BTB) have positively evaluated and approved this research.

### 2.2. Tissue Digestion and Thymic Stromal Cell Isolation

The thymuses were cleaned from blood clots and fatty tissue, weighed and chopped into small pieces. A single cell suspension was obtained by following a modified digestion protocol by Stoeckle [10]. Briefly, tissue fragments were digested during four incubation cycles with 1 mg/mL collagenase A (Worthington Biochemical Corporation, Lakewood, NJ, USA) and 0.1 mg/mL DNase I (Roche, Basel, Switzerland) in αMEM medium (Gibco, Thermo Fisher Scientific, Waltham, MA, USA), each lasting 15 min at 37 °C. Following the collagenase treatment, we incubated the tissue further, this time for 15 min with Versene (Lonza, Basel, Switzerland). As stem cells reside deep within the tissues, we used the cells obtained in the final digestion for further analysis. We counted the cells obtained from thymus digestion using trypan blue staining after each digestion cycle and collagenase change. The cells from the final digestion cycle (unenriched fraction), as well as the enriched cells after depletion and prior to FACS analysis, showed >95% viability in every thymus analyzed.

### 2.3. Thymic Stromal Cell Enrichment

The cells obtained during the final digestion cycle were washed and then labeled for 30 min with anti-human- CD3, CD4, CD8a, CD11b, CD14, CD16, CD19, CD56 (eBioscience, San Diego, CA, USA). The cells were then incubated for 30 min with Dynabeads goat anti-mouse IgG (Life Technologies, Carlsbad, CA, USA) and separated using a magnet. The obtained enriched stromal population of cells was used in all further experiments.

### 2.4. Spheroid Culture

The obtained enriched populations were grown in ultralow attachment flasks (Corning, New York, NY, USA) in a slightly modified medium for sphere cultivation, previously described by Ucar et al., 2014 [7]. αMEM (Gibco Thermo Fisher Scientific, Waltham, MA, USA) was supplemented with B27 supplement (Gibco, Thermo Fisher Scientific, Waltham, MA, USA), 0.5 µg/mL hydrocortisone (Sigma, St. Louis, MO, USA), 5 µg/mL insulin (Gibco, Thermo Fisher Scientific, Waltham, MA, USA), 4 µg/mL heparin (Sigma-Aldrich, St. Louis, MO, USA), 20 ng/mL bFGF (Peprotech, Thermo Fisher Scientific, Waltham, MA, USA) and 20 ng/mL EGF (Peprotech). The density of the plated cells was 10^5^ enriched cells/mL. We monitored the cultures for spheroid growth and fresh medium added every 3 days. The biological differences between samples were assessed by counting spheroids plated in quintuplicates in a 96-well plate 13 days after plating. The significance of the differences in sphere numbers between groups we determined by unpaired *t*-test or one-way ANOVA.

For the single-cell origin assay, enriched cells, obtained in the previously described manner, were mixed with cold (liquid) Mebiol^®^ gel (Cosmo Bio USA, Carlsbad, CA, USA) dissolved into the medium. An amount of 50 μL of the cell suspension was then plated in one well of a low attachment 96-well plate (Corning, New York, NY, USA) warmed to 37 °C. The temperature of the plate caused the gel to solidify and thus immobilize cells suspended in it. The gel was then overlaid with 150 μL of medium and the cells were cultured for 13 days prior to counting and photographing.

To assess the reformation ability of the sphere cells, we collected the spheres on a 20 µm strainer (pluriSelect, Leipzig, Germany) and dissociated them by incubating for 25 min with Versene (Lonza, Basel, Switzerland). The cells were then counted, replated in medium and monitored for reformation. The procedure was repeated every week as secondary cultures.

### 2.5. Growth Factor Treatment

The influence of Interleukin-22 (Il-22) and Cyr61 was assessed by adding the growth factors to primary and secondary spheroid cultures. Il-22 was used at 1, 2.5 and 5 ng/mL, while Cyr61 was used at 1 and 2.5 µg/mL. When used together, Il-22 was used at 5 ng/mL and Cyr61 at 2.5 µg/mL. Concentrations of 2 × 10^4^ of enriched cells (primary culture) or sphere cells (secondary cells) were plated in triplicates in 96-well plates. Spheroids were grown for 13 days when they were counted and photographed. Spheroid size was determined by ImageJ software (version 1.52a, National Institute of Health, Bethesda, MD, USA) [11]. The significance of the differences between treatments was determined by unpaired *t*-test or one-way ANOVA.

### 2.6. Limiting Dilution Assay

In order to determine the stem cell frequency in the thymus, as well as in the formed spheres, enriched cells (starting from 2 × 10^4^), unenriched cells (starting from 10^6^) and sphere cells (starting from 2 × 10^4^) were plated in quintuplicates in a two-fold series of dilutions, with dilutions positive for spheroids checked 13 days later. The series of dilutions were analyzed using the extreme limiting dilution analysis (ELDA) software (Version 4.7.1.2571 vom 16.01.2014) [12]. The interval of active cell frequency was calculated for at least three independent experiments.

### 2.7. 2′-Deoxyguanosine Treatment

We sought to optimize the stem cell isolation protocol and to simultaneously remove the bulk of thymocytes, so we utilized 2′-deoxyguanosine, previously known to selectively kill developing T-cells [13]. The thymic tissue was cleaned and prepared as previously described and then placed on a 40 μm filter (MiliporeSigma, Burlington, MA, USA) laid on a gelatinous sponge (Gelfoam, Pfizer, New York, NY, USA) immersed in αMEM medium (Gibco, Thermo Fisher Scientific, Waltham, MA, USA) containing 5% FCS (Gibco, Thermo Fisher Scientific, Waltham, MA, USA) and 1.35 mM 2′-deoxyguanosine (Sigma, St. Louis, MO, USA). A control tissue sample was incubated in the same manner but without 2′-deoxyguanosine. The tissue was incubated for 2 and 6 days, followed by digestion as described. The cells obtained by digestion were counted and their viability determined by using trypan blue staining. Although the cell viability obtained after digestion of fresh tissue was >95%, there was a decrease in viability after incubation, either with or without 2′-dGuo (consistently decreased to about 30% viable).

We plated the cells from the final digestion cycle at 2 × 10^4^ cells/mL and the number of spheres assessed 13 days later.

To see whether 2′-deoxyguanosine could hasten sphere formation and enhance their size, we incubated cells in spheroid medium as previously described, but with the addition of 1.35 mM 2′-deoxyguanosine, and analyzed for sphere formation and growth. The treated spheres and controls were counted 13 days after plating.

### 2.8. Multiplex PCR

Spheroids were collected 21 days after plating and lysed by snap freezing. RNA was transcribed into cDNA with transcript-specific primers by using MMLV reverse transcriptase (Applied Biosystems, Thermo Fisher Scientific, Waltham, MA, USA). The first PCR reaction was performed with a Taq polymerase (Sigma, St. Louis, MO, USA) during 15 cycles in a single tube with a mix of F1 and R primers, while the second PCR reaction was performed in separate tubes for each gene (F2 and R primers) for 42 cycles. The reaction was semi nested with common R primers. All of the primers were designed by Primer-BLAST and checked for specificity and dimer formation in silico and with known samples (Appendix A Table A1) [14]. At least one of the primers spans an exon–exon junction. The method was adapted for a small number of cells from the single cell multiplex PCR by Monteiro et al. (2004) [15,16].

### 2.9. Flow Cytometry

Spheroids were dissociated with Versene (Lonza Basel, Switzerland) and stained with mouse anti-human primary antibodies for 20 min, after which they were analyzed on FACSCalibur flow cytometer (Becton Dickinson BD, Franklin Lakes, NJ, USA). The antibodies used were antiCD44, (Tonbo Biosciences, San Diego, CA, USA), anti HLA-DQ, DP, DR (Becton Dickinson, BD), KRT8 (Becton Dickinson BD), KRT14 (Merck, Darmstadt, Germany), CD24 (eBioscience San Diego, CA, USA), EpCAM (Exbio, Praha, Czech Republic) and CD205 (eBioscience San Diego, CA, USA). The obtained results were processed with FlowJo version 10 (Becton Dickinson Company, Franklin Lakes, NJ, USA) and Weasel version 2.3 software, https://www.frankbattye.com.au/about.html.

### 2.10. Fluorescent and Confocal Microscopy

Spheroids were fixed and permeabilized with ice cold methanol (for intracellular antigens; KRT7/8) or BD Cytofix/Cytoperm (for surface antigens; CD205, CD44 and HLA DQ, DP, DR) for 20 min when unspecific binding was blocked for 20 min with Image-iT™ FX Signal Enhancer ReadyProbes™, Thermo Fisher Scientific, Waltham, MA, USA). After overnight incubation, the spheroids were stained with antibodies for an hour followed again by two washing cycles. The antibodies used were anti CD205 (eBioscience San Diego, CA, USA), rat anti-human CD44 (FITC, Tonbo Biosciences, San Diego, CA, USA), mouse anti-human KRT7/8 (Becton Dickinson, BD, Becton Dickinson BD, Franklin Lakes, NJ, USA) and mouse anti-human HLA-DQ, DP, DR (FITC, BD Becton Dickinson BD, Franklin Lakes, NJ, USA), while the secondary antibodies used Goat Anti-Mouse lgG Alexa Fluor™ 488 and Alexa Fluor™ 647 conjugates (Thermo Fisher Scientific, Waltham, MA, USA). Nuclei were stained with NucBlue™ Live ReadyProbes™ Reagent, Thermo Fisher Scientific, Waltham, MA, USA) for 30 min. Spheroids were suspended in 50 µL of PBS with 2% FCS and immobilized on glass slides by centrifugation.

## 3. Results

### 3.1. Spheroid Formation Potential of Cells from Human Thymus

**Figure 1:**
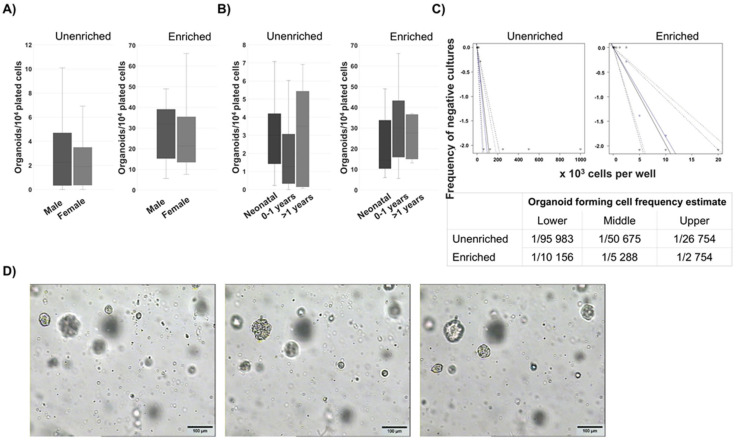
Spheroid number, biological differences between samples and single-cell origins of spheroids. (**A**) Enriched cells (2 × 10^4^ cells/mL) and unenriched cells (10^5^ cells/mL) were plated in quintuplicates in order to assess the stemness potential of biological samples and the increase in the number of spheres as depicted in a box and whisker plot. An amount of 44 samples of unenriched (*n* = 44) and 38 of enriched cells (*n* = 38) were analyzed according to the patients’ sex. (**B**) The number of spheroids was analyzed according to the age of the patients. An amount of 43 patient samples (*n* = 43) were analyzed for unenriched and 36 for enriched cells (*n* = 36) with the smallest number of patients per category being 7. The distribution of sphere numbers is depicted as a box and whisker plot. (**C**) Cells were plated in quadruplicates in two-fold series of dilutions and the dilutions positive for spheroids were analyzed 13 days later. The results were analyzed by ELDA and the frequency of negative cultures presented as well as the calculated frequencies of spheroid-forming cells. The experiments were repeated three times for each group (*n* = 3). (**D**) Enriched cells (2 × 10^4^) were plated in liquid Mebiol^®^ gel which was then allowed to solidify. Spheroids were formed from individual cells as seen in different focal lengths on the photographs. The bar represents 100 nm; 250× magnification. The experiment was repeated with five biological samples (*n* = 5).

Cells with spheroid forming potential were reliably isolated from human thymus samples and discernible spheroids were formed, on average, 7 days after plating. Based on the ability to form spheroids, the progenitor/stem cell presence was not limited by sex (Figure 1A) or age (from neonatal to 10 years of age) (Figure 1B) of the patient, as the spheroids were present in all of the samples analyzed, indicating a resident progenitor population. Interestingly, a larger variance in stem cell potential was found in the thymuses of patients older than one year. The stem cell frequency was determined by limiting dilution assay and it was estimated to be one in 50,675 unenriched thymic cells and one in 5288 enriched cells. These data confirm our method of stem cell enrichment (Figure 1C). Culturing cells in a semi-solid medium which inhibited cell aggregation yielded spheroids and showed that they were, indeed, derived from single cells (Figure 1D).

### 3.2. Reformation Ability of Cells from Human Thymus

**Figure 2:**
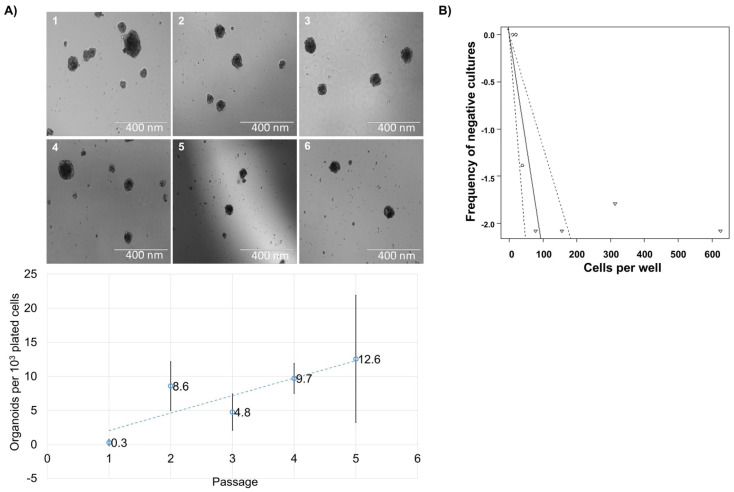
Spheroid reformation and limiting dilution assay of the cells. (**A**) Spheroids were dissociated and replated up to 6 times from a single sample. The spheroids were photographed at 160× magnification and the bar represents 400 nm. (**B**) Cells obtained by spheroid dissociation were plated in a series of dilutions and analyzed by ELDA 13 days later. The circle represents the log value of the proportion of negative cultures in a multiplicate and the number of cells in those cultures. The triangle represents the number of cells where there are no negative cultures in a multiplicate. The interval of the frequency of negative cultures with a confidence interval of 95% was computed. The experiment was undertaken with two biological samples (*n* = 2). The number of spheroids per every 10^3^ cells plated and the number of spheroids for each sample and passage are presented. The experiment was performed with spheroids obtained from 2 biological samples.

One of the main prerequisites of classifying a cell as a progenitor or precursor is the ability to self-renew. To test this ability, we enzymatically dissociated spheroids and replated the obtained cells. In all examined instances, cells reformed new spheroids (Figure 2A). This dissociation was repeated up to six times (up to the 7th spheroid) but it was noticed that the total cell number declined. This observation led us to believe that not all of the cells within the spheroid retain the true self-renewal capacity of progenitor/stem cells. We performed a limiting dilution assay with cells from dissociated primary spheroids, and found that, in secondary spheroids, one in every 43.2 sphere cells has the capability of reformation (Figure 2B). Even though the cell numbers declined with reformation, the number of spheres per 10^3^ cells plated increased, albeit irregularly (Figure 2). We postulate that the cells formed by the division of the initial spheroid-forming cell are likely directed towards terminal differentiation and thus lose the ability to divide independently of cell–cell or cell–matrix contact. 

### 3.3. Expressional Analysis of Thymus Spheroid Cells

**Figure 3:**
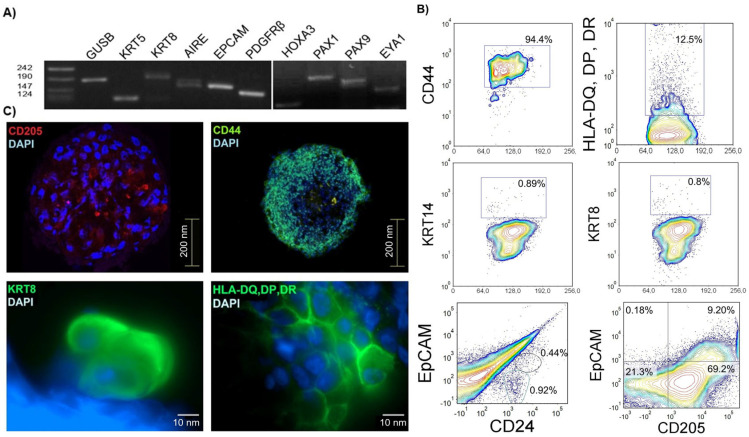
Gene and protein expression of cells from spheroids. (**A**) Gene expression in spheroid cells detected by multiplex PCR. Expression of genes typical for mature thymic epithelial cells, *KRT5*, *KRT8*, *AIRE* and *EpCAM*; genes present during thymic organogenesis, *HOXA3*, *PAX1*, *PAX9* and *EYA1*; and for mesenchymal cells *PDGFRβ*. The experiment was repeated with spheroids from 4 different biological samples (*n* = 4). (**B**) Protein expression analyzed by flow cytometry. Dead cells, residual lymphocytes from culture and cell debris were excluded from analysis based on CD45 expression, size and granularity. CD44 (upper left), HLA class II (upper right, 12.5%), KRT8 (middle left, 0.9%), KRT14 (middle right, 0.8%), EpCAM, and CD24 (bottom left) were expressed on a small population of cells (0.44%); EpCAM and CD205 (bottom right) were expressed on 9.2% cells from spheroids (*n* = 3). (**C**) Protein expression analyzed by confocal and fluorescent microscopy. Staining with specific antibodies; top row: left: CD205; right: CD44; second row: left: KRT8; right: HLA-DQ, DP, DR. DAPI: staining of nuclei. Photographs were taken on an Olympus FV3000 and Zeiss fluorescent microscope. The scale bars represent 200 nm (upper row) and 10 nm (lower row) (*n* = 3).

Deducing from cell enrichment and limiting dilution experiments, we hypothesized that the spheroid-initiating cells reside within the non-hematopoietic population of the thymus. To determine their precise identity, we reverse transcribed and amplified mRNA from spheroids by multiplex PCR [16], thus insuring amplification specificity from limited material (Figure 3A). Spheroids proved positive for genes vital for thymus function. The expression of *EpCAM* confirmed the presence of differentiated epithelial cells. Specific keratins show the presence of medullary (*KRT5*) and cortical (*KRT8*) thymic epithelial cells [17,18]. We also detected the expression of *AIRE*, a transcription factor found to be fundamental for the proper expression of tissue-specific antigens by medullary epithelial cells [19,20]. We also analyzed the expression of FOXN1 in spheroid samples from different thymuses and have indeed detected the expression of FOXN1. However, the expression was not consistent in all samples. To check whether mesenchymal cells contribute to spheroids, we analyzed the expression of the platelet-derived growth factor receptor beta (*PDGFRβ*), found on a variety of cells but mostly on those of mesenchymal origin [21]. Interestingly, some spheroids were positive for PDGFRβ.

Studies on mouse thymic organogenesis have provided insight into the molecular mechanisms involved and have defined the genes indispensable for the proper development and organization of the thymic stromal compartment—*HOXA3*, *PAX1*, *PAX9* and *EYA1*. The expression of these genes has been found to be conserved between mice and humans, with PAX1 having an even more restricted expression pattern in humans [22]. All of the aforementioned genes were found to be expressed in thymic spheroids obtained from the postnatal human thymus. These data strongly suggest that a population of immature cells should be capable of maintaining thymic stroma.

The protein expression was analyzed by flow cytometry (Figure 3B) and by confocal and fluorescence microscopy (Figure 3C). It was shown that the spheroid cells are positive for CD44, a glycoprotein expressed on a wide variety of cells. HLA-DQ, DP, and DR, detected by the common antibody for MHC II molecules and expressed on antigen presenting cells, were found to be present on 12.5% of spheroid cells [23]. Mature thymic epithelial cells were detected by KRT8 (cTEC) and KRT14 (mTEC) and were found to compose <1% of spheroid cells [18]. Cells positive for KRT8 were also found by fluorescent microscopy. CD205 was found to be expressed on 80% of the spheroid cells. Flow cytometry revealed that it was expressed with EpCAM (9.3%) and CD24 (9.4%). The two populations were mutually exclusive. EpCAM was positive on a small population of cells, confirming that the mature thymic epithelial cells represented a minority of spheroid cells. CD24 was considered as a possible specific progenitor marker due to its expression by human intestinal epithelial stem cells as well as by a number of cancer stem cells [24,25]. CD24 was found on 1.4% of spheroid cells. 

### 3.4. 2-Deoxyguanosine Treatment of the Stromal Cell Population

**Figure 4:**
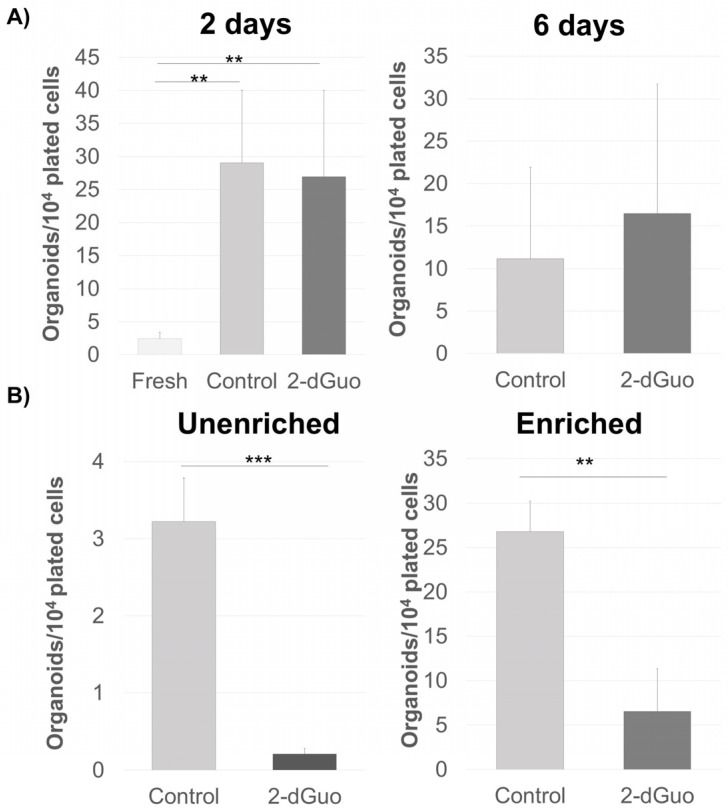
2′-deoxyguanosine effect on a spheroid yield. (**A**) Thymic tissue incubated with and without 2′-dGuo for 2 and 6 days when it was digested and plated. The number of spheroids was assessed 13 days later and compared with those obtained from tissue digested on arrival. Experiments were repeated three times (*n* = 3) for shorter incubation and two times (*n* = 2) for longer. The average number of spheroids per 10^4^ plated cells ± standard deviation in every category shown. (** *p* < 0.01). (**B**) 2′-dGuo was added to the spheroid growth medium, unenriched cells were plated and the number of spheroids was assessed 13 days later. The experiment was repeated with four different patient samples (*n* = 4). The average number of spheroids per 10^4^ plated cells ± standard deviation in every category is depicted on the graph (** *p* < 0.01, *** *p* < 0.001).

To enrich the stromal cell population, and consequently the population of spheroid-forming cells, we utilized the property of 2′-deoxyguanosine (2-dGuo) to selectively kill T lymphocytes [13,26]. As the goal of different treatments was to increase the number of progenitors, our final readout was the number of spheroids formed under each treatment condition, as shown in Figure 4 for 2-dGuo. Shorter tissue incubation (2 days) with 1.35 mM 2-dGuo led to a significant increase in the number of spheroids formed in the final digestion cycle when compared with fresh tissue, but their number was comparable to those of cells grown without 2-dGuo. Extending the incubation period to six days led to a moderate increase in the number of spheroids formed, possible because of the tissue degradation during such prolonged incubation (Figure 4A). The outcome of the spheroid formation was also found to be dependent on the patient sample. We sought to ascertain whether this effect was due, as suspected, to the depletion of thymocytes from the tissue leading to the increased percentage of stromal cells in the final fraction or due to a stimulatory influence of 2-dGuo. We plated unenriched cells obtained from fresh tissue in spheroid medium with the addition of 1.35 mM 2-dGuo. We found that the direct presence of 2-dGuo in the medium inhibited spheroid formation (an 11- to 21.8-fold decrease in spheroid number) (Figure 4B). Therefore, the continuous exposure of cells to 2-dGuo had an unfavorable effect on the proliferative capabilities of the precursors. It cannot be excluded that cell debris from apoptotic T lymphocytes had an adverse effect on the growth of other cells in the culture.

### 3.5. Cyr61 and Interleukin-22 Impact on Spheroid Formation

To enhance the culture conditions of spheroid-forming cells, we cultured primary thymus stromal cells and dissociated spheroids (secondary cells) with a factor discovered to be implicated in the thymus epithelial cell expansion in mice, Cysteine-rich protein 61 (Cyr61) [27]. We also tested a cytokine which has been known to positively influence the proliferative capabilities of a variety of tissue-resident stem cells, Interleukin-22 (Il-22) [28,29,30].

**Figure 5:**
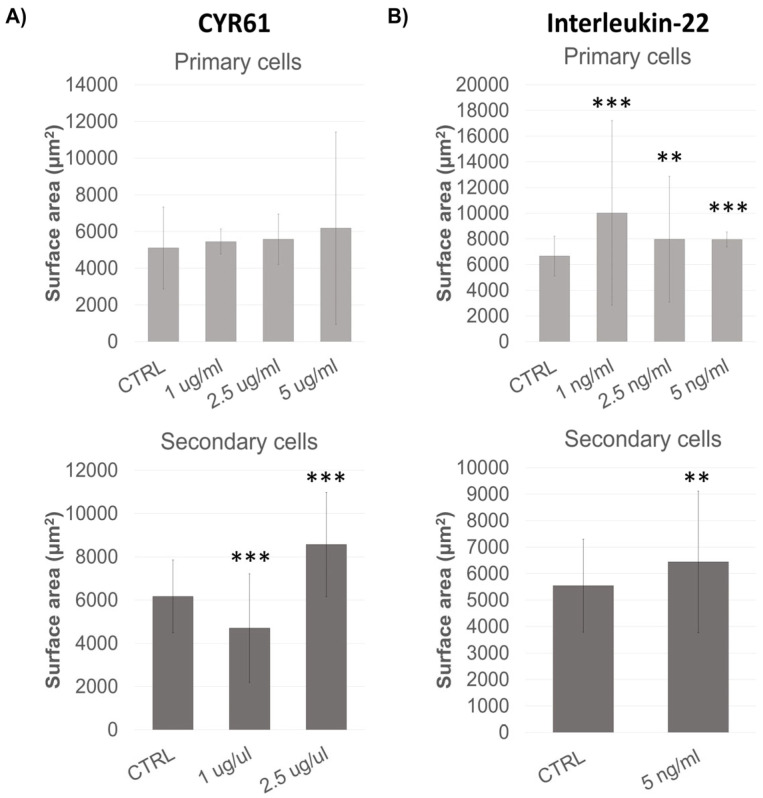
The effect of Cyr61 and Il-22 on spheroid number and surface area. Primary cells and dissociated spheroids (secondary cells) were grown for 13 days with growth factors, counted and photographed in order to determine their size. Spheroid surface area was analyzed by ImageJ version 1.52a and is expressed in μm^2^. The mean surface area and standard deviation for all photographed spheroids of untreated controls and treated samples is plotted on the graph. A minimum of 20 spheroids was analyzed for each control or treatment. (**A**) Cyr61 was used in concentrations of 1, 2.5 and 5 μg/mL for primary cells, and 1 and 5 μg/mL for secondary cells (*** *p* < 0.0001). (**B**) Interleukin-22 (Il-22) was used at 1, 2.5 and 5 ng/mL with primary cells and 5 ng/mL with secondary cells (*** *p* < 0.0001; ** *p* < 0.001).

We found that Cyr61 boosts the rate of spheroid cell divisions and influences the size redistribution in favor of larger spheroids, but these effects seem to be dependent on the patient sample. Cyr61 at 2.5 μg/mL impacted the proliferation of dissociated spheroid cells, but only in some samples and therefore was not found to be significant (Figure 5A).

The treatment of primary thymus cells with Il-22 (1 and 2.5 ng/mL) increased spheroid number and size, but only in some patient samples (Figure 5B).

When cells were treated simultaneously with 2.5 μg/mL Cyr61 and 2.5 ng/mL Il-22, no significant effect on spheroid size or number was seen. 

## 4. Discussion

Up to today there have been many different attempts to establish thymus-like conditions for T-cell differentiation. The first models used immunocompromised mice engrafted with human fetal thymus and liver tissue fragments and in vitro models with human–mouse chimeric fetal thymic organ cultures [31,32,33]. Montel-Hagen [34] generated artificial thymic spheroids (ATO) from human and murine hematopoietic stem and progenitor cells cultivated with murine stromal cells expressing Notch ligand. Many of the established models needed feeder layers or to prove their functionality in vivo.

In parallel, there were many attempts to cultivate and differentiate thymic stem cells which could reconstitute the thymus conditions in vitro. Most of these experiments were undertaken on murine thymic tissue. Bleul et al. established functional thymus initiated by a postnatal epithelial progenitor cells [35]. Furthermore, the existence of a bipotent epithelial progenitor population was identified in the murine adult thymus, and is capable of differentiation into both cortical and medullar epithelial cells. The identified progenitor cells were EpCAM and FOXN1+ [36,37]. Ocampo-Godinez et al. [38] established the culture of functional TECs and thymic interstitial cells from mice by cultivating them in DMEM/F-12 medium with 20% FBS. These cells were able to form thymospheres and expressed functional markers for T-cell development, such as Aire, Il25, CD80 and CCL21. Lim et al. [39] isolated functional thymic epithelial spheroids from adult murine thymus. These consisted of epithelial cells and were able to proliferate in a complex medium sustaining Wnt- and EGF-dependent signaling. They contained epithelial progenitors, which could be differentiated either in cTEC or mTEC. Hubscher et al. [40] managed to grow mouse TEC spheroids, which mediate T-cell development. They grew cells in defined spheroid-based medium on matrix-based hydrogel with the addition of FGF7. The culture conditions allowed spheroid formation from neonatal, but not adult TECs. On the other hand, there were numerous experiments in which thymic cells were differentiated from human and mouse embryonic stem cells or induced pluripotent stem cells. Additionally, iTEC cell-induced thymic epithelial cells were produced by direct reprogramming of mouse fibroblasts by FOXN1 [41]. Tetteh et al. [42] showed that neural crest-derived mesenchymal cells supported thymic reconstitution after lethal irradiation in mice.

Recently Campinoti et al. [8] identified epithelial cells in human thymuses which had the epithelial–mesenchymal phenotype and were capable of long-term proliferation and establishing a functional microenvironment for human T-cell differentiation. However, there are still different hypotheses about the origin of thymic stem cells, whether they are of epithelial or mesenchymal origin, and, accordingly, their markers. For these reasons, we approached the issue from a different angle and, considering the unknown phenotype of precursors, removed hematopoietic cells. Our efforts at improving the isolation and culture technique with 2-deoxyguanosine, Interleukin-22 and Cyr-61 did not provide definitive results. Tissue treatment with 2-dGuo yielded a greater proportion of stromal cells within the population obtained by digestion, but incubation is a lengthy process and the addition of 2-dGuo to the cell medium had a negative effect on spheroid formation. Interestingly, interleukin-22 and Cyr61 treatment had highly individualized effects. It could be possible that the human thymus contains several different types of stem/progenitor cell populations with different responses to the compounds used. Thus, the differing effects could be due to the varying proportions of spheroids derived from different stem cells.

By growing cells of the postnatal human thymus in low attachment conditions with the addition of growth factors [7], we continuously obtained 3D clusters of cells i.e., spheres or spheroids. Due to the presence of cells with phenotypic markers peculiar to mesenchymal cells, which are known for their high migratory potential, we needed to test whether the cells were of single-cell origin or cell aggregates by immobilizing them in a semi-liquid gel. However, more experiments are needed to completely prove that there was no cell migration present in our experimental conditions and test the proliferative activity of the cells in culture. Presuming that each sphere was formed from a single stem cell, the stem cell potential of postnatal human thymuses was shown to be independent of the age or sex of the patient. However, the differences between individual samples became bigger in samples from patients older than one year. The limited dilution assay shows that this sphere-forming population is rare, as stem cells usually are. A population of cells within the clusters has also been shown to possess the capacity for self-renewal. However, the cells that originated from the sphere-forming progenitors did not always retain self-renewal properties. Nonetheless, these findings together indicate the existence of stromal progenitors of the human thymus. Cells from the clusters express a plethora of markers specific for early (HOXA3, EXA1, PAX1 and PAX9) and late (EpCAM, KRT7/8, KRT14) thymic epithelial differentiation and epithelial cell function (HLA-DQ, DP, DR and AIRE) [18,20,22,23]. HOXA3 is expressed in the third pharyngeal pouch during embryonic development and its deletion leads to the inability of the pouch to differentiate into the thymus and parathyroid glands [43]. EYA1 is expressed in the same context and with the same consequences as HOXA3, while PAX1 and PAX9 continue the signaling cascade during thymic organogenesis [44]. The expression of these early genes reveals the presence of an extremely immature population of cells within the postnatal thymus. The expression of functionally important genes, such as HLA-DQ, DP, DR and AIRE, shows that some of the cells within the clusters have differentiated into competent keratinocytes. C205, which we found to be present on spheroid cells, is also expressed on antigen presenting cells, more specifically, on cortical epithelial cells and dendritic cells of the thymus [45]. The abundance of CD205 cells in our thymus cultures may suggest that the spheroids are predominantly cortical epithelium rather than medulla epithelium. We cannot rule out the possibility that our culture conditions favor cTEC development over mTEC development. Interestingly, CD44 and PDGFRβ, the markers more commonly connected to mesenchymal cells, were found to be abundantly present in the clusters [46]. CD24, a glycoprotein found on mesenchymal bone marrow stem cells, but also on intestinal epithelial stem cells and hematopoietic cells, was found on a small population within the clusters [47]. Taken together, our results are in accordance with those obtained by Campinoti et al. [8], showing that the human thymus contains epithelial–mesenchymal hybrid cells capable of expansion and differentiation into functional thymic stromal cells. We chose to call our clusters spheroids because of the heterogeneity shown. In view of the possibility that the cells’ presumed progenitors have a mixed epithelial–mesenchymal phenotype, it would be interesting to observe the localization of signals from epithelial and mesenchymal markers in spheroids simultaneously. Taking into account the differential response of spheroid-forming cells to Cyr61 and Il-22, and differences in spheroid-forming potential between patient samples, we propose the existence of two distinct populations of tissue-resident progenitor/stem cells in the postnatal human thymus. Their distinctions, specificities and precise roles in thymic stromal maintenance are yet to be determined. The future of the research described in this paper surely lies in a single cell approach and cell tracking, including the precise analysis of signals within the stem cell. In this paper we have described the very robust method that we used and the cells we obtained. The population we isolated and described here is ideal for a more thorough examination with antibodies to markers of mesenchymal phenotype, and this is the matter of our future work.

## Data Availability

The original contributions presented in this study are included in the article. Further inquiries can be directed to the corresponding author.

## Appendix A

**Table A1:** Multiplex primer sequences. The first reaction was performed by using the mixture of F1 and R primers, after which the specific genes were amplified separately with F2 and R primers. All of the primers were specifically designed to bind to cDNA, at least one of the primers spans an exon–exon junction.

Gene	Sequence
GUSB	F1: ACTTCTCTGACAACCGACGC
	F2: GTACGAACGGGAGGTGATCC
	R: CATTCACCCACACGATGGCA
KRT5	F1:AGGGCGAGGAATGCAGACTC
	F2: TGGACCAGTCAACATCTCTGT
	R:ACTGCTACCTCCGGCAAGA
KRT8	F1:GCTCCAGGCTGAGATTGAGG
	F2:TAAGGATGCCAACGCCAAGT
	R: CTCAGACCACCTGCATAGCC
AIRE	F1:ACTTCTGGAGGGTGCTGTTC
	F2: AGACTCCCCACCAAGAGGAA
	R: CCTTCAGTTGAGAGCCTGGG
EpCAM	F1: AGAGCAAAACCTGAAGGGGC
	F2: TCCATGTGCTGGTGTGTGAA
	R: TCTGAAGTGCAGTCCGCAAA
PDGFRB1	F1: CCCTTCCTCCATCCCTCTGT
	F2: GGAGAGGGCAGTAAGGAGGA
	R: GAAGCCGCATGGTGTCCTTG
HOXA3	F1: GGCAAGGACACAGCATCTTG
	F2: GACACCGGAAAAGGCGATTC
	R: CCCGCGCAGACCTAGAAAGA
PAX1	F1: GCTGCCCTACAACCACATCT
	F2: TGGGCATCCGGACGTTTATG
	R: GGAGGCCGACTGAGTGTATTTA
PAX9	F1: CTTTTAGGGCGTGTCCCCAG
	F2: CTTTCATCGGGGCACAGACT
	R: AAGGCTGGCTCCATTGCT

## References

[1] Boyd R.L., Tucek C.L., Godfrey D.I., Izon D.J., Wilson T.J., Davidson N.J., Bean A.G.D., Ladyman H.M., Ritter M.A., Hugo P. (1993). The Thymic Microenvironment. Immunol. Today.

[2] Kumar B.V., Connors T.J., Farber D.L. (2018). Human T Cell Development, Localization, and Function throughout Life. Immunity.

[3] Steinmann G.G., Klaus B., Muller-Hermelink H.K. (1985). The Involution of the Ageing Human Thymic Epithelium Is Independent of Puberty. A Morphometric Study. Scand. J. Immunol..

[4] Palmer S., Albergante L., Blackburn C.C., Newman T.J. (2018). Thymic Involution and Rising Disease Incidence with Age. Proc. Natl. Acad. Sci. USA.

[5] Nijhuis E.W., Nagelkerken L. (1992). Age-Related Changes in Immune Reactivity: The Influence of Intrinsic Defects and of a Changed Composition of the CD4+ T Cell Compartment. Exp. Clin. Immunogenet..

[6] Chaudhry M.S., Velardi E., Dudakov J.A., van den Brink M.R.M. (2016). Thymus: The Next (Re)Generation. Immunol. Rev..

[7] Ucar A., Ucar O., Klug P., Matt S., Brunk F., Hofmann T.G., Kyewski B. (2014). Adult Thymus Contains FoxN1- Epithelial Stem Cells That Are Bipotent for Medullary and Cortical Thymic Epithelial Lineages. Immunity.

[8] Campinoti S., Gjinovci A., Ragazzini R., Zanieri L., Ariza-McNaughton L., Catucci M., Boeing S., Park J.E., Hutchinson J.C., Muñoz-Ruiz M. (2020). Reconstitution of a Functional Human Thymus by Postnatal Stromal Progenitor Cells and Natural Whole-Organ Scaffolds. Nat. Commun..

[9] Sheridan J.M., Keown A., Policheni A., Roesley S.N.A.A., Rivlin N., Kadouri N., Ritchie M.E., Jain R., Abramson J., Heng T.S.P.P. (2017). Thymospheres Are Formed by Mesenchymal Cells with the Potential to Generate Adipocytes, but Not Epithelial Cells. Cell Rep..

[10] Stoeckle C., Rota I.A., Tolosa E., Haller C., Melms A., Adamopoulou E. (2013). Isolation of Myeloid Dendritic Cells and Epithelial Cells from Human Thymus. J. Vis. Exp..

[11] Schneider C.A., Rasband W.S., Eliceiri K.W. (2012). NIH Image to ImageJ: 25 Years of Image Analysis HHS Public Access. Nat. Methods.

[12] Hu Y., Smyth G.K. (2009). ELDA: Extreme limiting dilution analysis for comparing depleted and enriched populations in stem cell and other assays. J. Immunol. Methods.

[13] Ramsdell F., Zúñiga-Pflücker J.C., Takahama Y. (2006). In Vitro Systems for the Study of T Cell Development: Fetal Thymus Organ Culture and OP9-DL1 Cell Coculture. Curr. Protoc. Immunol..

[14] Ye J., Coulouris G., Zaretskaya I., Cutcutache I., Rozen S., Madden T.L. (2012). Primer-BLAST: A Tool to Design Target-Specific Primers for Polymerase Chain Reaction. BMC Bioinform..

[15] Monteiro M., Rocha B., Veiga-fernandes H. (2004). Quantification of Multiple Gene Expression in Individual Cells. Genome Res..

[16] Horvat L., Skelin J., Jelić Puškarić B., Feliciello I., Heckel D., Madunić J., Kardum-Skelin I., Matulić M., Radić-Krišto D., Antica M. (2018). Notch pathway connections in primary leukaemia samples of limited size. Transl. Med. Commun..

[17] Shezen E., Okon E., Ben-Hur H., Abramsky O. (1995). Cytokeratin Expression in Human Thymus: Immunohistochemical Mapping. Cell Tissue Res..

[18] Lee E.N., Park J.K., Lee J.-R., Oh S.-O., Baek S.-Y., Kim B.-S., Yoon S. (2011). Characterization of the Expression of Cytokeratins 5, 8, and 14 in Mouse Thymic Epithelial Cells during Thymus Regeneration Following Acute Thymic Involution. Anat. Cell Biol..

[19] Nagamine K., Peterson P., Scott H.S., Kudoh J., Minoshima S., Heino M., Krohn K.J., Lalioti M.D., Mullis P.E., Antonarakis S.E. (1997). Positional Cloning of the APECED Gene. Nat. Genet..

[20] Perniola R. (2018). Twenty Years of AIRE. Front. Immunol..

[21] Veevers-Lowe J., Ball S.G., Shuttleworth A., Kielty C.M. (2011). Mesenchymal Stem Cell Migration Is Regulated by Fibronectin through A5β1-Integrin-Mediated Activation of PDGFR-β and Potentiation of Growth Factor Signals. J. Cell Sci..

[22] Farley A.M., Morris L.X., Vroegindeweij E., Depreter M.L.G., Vaidya H., Stenhouse F.H., Tomlinson S.R., Anderson R.A., Cupedo T., Cornelissen J.J. (2013). Dynamics of Thymus Organogenesis and Colonization in Early Human Development. Development.

[23] Unanue E.R., Turk V., Neefjes J. (2016). Variations in MHC Class II Antigen Processing and Presentation in Health and Disease. Annu. Rev. Immunol..

[24] Jaggupilli A., Elkord E. (2012). Significance of CD44 and CD24 as Cancer Stem Cell Markers: An Enduring Ambiguity. Clin. Dev. Immunol..

[25] Gracz A.D., Fuller M.K., Wang F., Li L., Stelzner M., Dunn J.C.Y.Y., Martin M.G., Magness S.T. (2013). Brief Report: CD24 and CD44 Mark Human Intestinal Epithelial Cell Populations with Characteristics of Active and Facultative Stem Cells. Stem Cells.

[26] Jenkinson E.J., Franchi L.L., Kingston R., Owen J.J.T. (1982). Effect of Deoxyguanosine on Lymphopoiesis in the Developing Thymus Rudimentin Vitro: Application in the Production of Chimeric Thymus Rudiments. Eur. J. Immunol..

[27] Emre Y., Irla M., Dunand-Sauthier I., Ballet R., Meguenani M., Jemelin S., Vesin C., Reith W., Imhof B.A. (2013). Thymic Epithelial Cell Expansion through Matricellular Protein CYR61 Boosts Progenitor Homing and T-Cell Output. Nat. Commun..

[28] Dudakov J.A., Hanash A.M., Jenq R.R., Young L.F., Ghosh A., Singer N.V., West M.L., Smith O.M., Holland A.M., Tsai J.J. (2012). Interleukin-22 Drives Endogenous Thymic Regeneration in Mice. Science.

[29] Feng D., Kong X., Weng H., Park O., Wang H., Dooley S., Gershwin M.E., Gao B. (2012). Interleukin-22 Promotes Proliferation of Liver Stem/Progenitor Cells in Mice and Patients with Chronic Hepatitis B Virus Infection. Gastroenterology.

[30] Lindemans C.A., Calafiore M., Mertelsmann A.M., O’Connor M.H., Dudakov J.A., Jenq R.R., Velardi E., Young L.F., Smith O.M., Lawrence G. (2015). Interleukin-22 Promotes Intestinal-Stem-Cell-Mediated Epithelial Regeneration. Nature.

[31] Plum J., De Smedt M., Defresne M.P., Leclercq G., Vandekerckhove B. (1994). Human CD34+ fetal liver stem cells differentiate to T cells in a mouse thymic microenvironment. Blood.

[32] McCune J.M., Namikawa R., Kaneshima H., Shultz L.D., Lieberman M., Weissman I.L. (1988). The SCID-hu mouse: Murine model for the analysis of human hematolymphoid differentiation and function. Science.

[33] Vandekerckhove B.A., Namikawa R., Bacchetta R., Roncarolo M.G. (1992). Human hematopoietic cells and thymic epithelial cells induce tolerance via different mechanisms in the SCID-hu mouse thymus. J. Exp. Med..

[34] Montel-Hagen A., Tsai S., Seet C.S., Crooks G.M. (2022). Generation of Artificial Thymic Organoids from Human and Murine Hematopoietic Stem and Progenitor Cells. Curr. Protoc..

[35] Bleul C.C., Corbeaux T., Reuter A., Fisch P., Mönting J.S., Boehm T. (2006). Formation of a Functional Thymus Initiated by a Postnatal Epithelial Progenitor Cell. Nature.

[36] Wong K., Lister N.L., Barsanti M., Lim J.M.C., Hammett M.V., Khong D.M., Siatskas C., Gray D.H.D., Boyd R.L., Chidgey A.P. (2014). Multilineage Potential and Self-Renewal Define an Epithelial Progenitor Cell Population in the Adult Thymus. Cell Rep..

[37] Ulyanchenko S., O’Neill K.E., Medley T., Farley A.M., Vaidya H.J., Cook A.M., Blair N.F., Blackburn C.C. (2016). Identification of a Bipotent Epithelial Progenitor Population in the Adult Thymus. Cell Rep..

[38] Lim S., J F van Son G., Wisma Eka Yanti N.L., Andersson-Rolf A., Willemsen S., Korving J., Lee H.G., Begthel H., Clevers H. (2024). Derivation of functional thymic epithelial organoid lines from adult murine thymus. Cell Rep..

[39] Ocampo-Godinez J.M., Gonzalez-Quiroz J.L., Cote-Palafox H., George E., Vergara-Lope Nuñez J.A., Villagomez-Olea G., Vazquez-Vazquez F.C., Lopez-Villegas E.O., Leon-Avila G., Dominguez-Lopez M.L. (2023). Primary explants of the postnatal thymus allow the expansion of clonogenic thymic epithelial cells that constitute thymospheres. Stem Cell Res. Ther..

[40] Hübscher T., Lorenzo-Martín L.F., Barthlott T., Tillard L., Langer J.J., Rouse P., Blackburn C.C., Holländer G., Lutolf M.P. (2024). Thymic epithelial organoids mediate T-cell development. Development.

[41] Bredenkamp N., Ulyanchenko S., O’Neill K.E., Manley N.R., Vaidya H.J., Blackburn C.C. (2014). An organized and functional thymus generated from FOXN1-reprogrammed fibroblasts. Nat. Cell Biol..

[42] Tetteh D.N., Isono K., Hikosaka-Kuniishi M., Yamazaki H. (2025). Neural Crest-Derived Mesenchymal Cells Support Thymic Reconstitution After Lethal Irradiation. Eur. J. Immunol..

[43] Chojnowski J.L., Masuda K., Trau H.A., Thomas K., Capecchi M., Manley N.R. (2014). Multiple Roles for HOXA3 in Regulating Thymus and Parathyroid Differentiation and Morphogenesis in Mouse. Development.

[44] Figueiredo M., Zilhão R., Neves H. (2020). Thymus Inception: Molecular Network in the Early Stages of Thymus Organogenesis. Int. J. Mol. Sci..

[45] Jiang W., Swiggard W.J., Heufler C., Peng M., Mirza A., Steinman R.M., Nussenzweig M.C. (1995). The Receptor DEC-205 Expressed by Dendritic Cells and Thymic Epithelial Cells Is Involved in Antigen Processing. Nature.

[46] Maleki M., Ghanbarvand F., Behvarz M.R., Reza Behvarz M., Ejtemaei M., Ghadirkhomi E. (2014). Comparison of Mesenchymal Stem Cell Markers in Multiple Human Adult Stem Cells. Int. J. Stem Cells.

[47] Fang X., Zheng P., Tang J., Liu Y. (2010). CD24: From A to Z. Cell. Mol. Immunol..

